# Exploiting the Diversity of the Heat-Shock Protein Family for Primary and Secondary Tauopathy Therapeutics

**DOI:** 10.2174/157015911798376226

**Published:** 2011-12

**Authors:** Jose F Abisambra, Umesh K Jinwal, Jeffrey R Jones, Laura J Blair, John Koren, Chad A Dickey

**Affiliations:** Department of Molecular Medicine, USF Health Byrd Alzheimer’s Institute, Tampa, FL 33613, USA

**Keywords:** Heat shock proteins, chaperones, neurodegeneration, alzheimer, Hsp27, Tau.

## Abstract

The heat shock protein (Hsp) family is an evolutionarily conserved system that is charged with preventing unfolded or misfolded proteins in the cell from aggregating. In Alzheimer’s disease, extracellular accumulation of the amyloid β peptide (Aβ) and intracellular aggregation of the microtubule associated protein tau may result from mechanisms involving chaperone proteins like the Hsps. Due to the ability of Hsps to regulate aberrantly accumulating proteins like Aβ and tau, therapeutic strategies are emerging that target this family of chaperones to modulate their pathobiology. This article focuses on the use of Hsp-based therapeutics for treating primary and secondary tauopathies like Alzheimer’s disease. It will particularly focus on the pharmacological targeting of the Hsp70/90 system and the value of manipulating Hsp27 for treating Alzheimer’s disease.

## INTRODUCTION

The chaperone system of heat shock proteins (Hsps) is an ancient and evolutionarily conserved protein family that regulates nascent folding as well as modulates the fate of unstructured or pathologically misfolded proteins, termed clients [1]. Misfolded or intrinsically unfolded clients of the chaperone network expose hydrophobic signatures that Hsps recognize and bind. Hsps attach to clients in these hydrophobic regions and, via a well-coordinated system of co-chaperone interactions, are routed for renaturation (if suitable) or degradation. 

Neurodegenerative disorders such as tauopathies are characterized by the pathological aggregation of misfolded proteins. Thus, Hsps have become a promising system that may serve as a platform to modulate disease processes where protein misfolding is a common feature. Insofar, there have been several promising attempts to manipulate Hsps by pharmacological interventions [2, 3] and gene-delivery approaches [4-10].

The aim of this review is to chronicle strides in the investigation of Hsps from the perspective of Alzheimer’s disease (AD), the most common tauopathy, where intracellular tangles and extracellular amyloid plaques are pathological hallmarks. In particular, this review will focus on pharmacological therapeutics and genetic interventions to modulate Hsp70 and Hsp90. Finally, the current findings on research involving both Hsp27 in AD will be presented. 

## ALZHEIMER’S DISEASE

Alzheimer’s disease is a chronic neurodegenerative disorder characterized by progressive memory loss. Currently,5.3 million people in the U.S. have AD. There are an estimated 4% of AD cases in persons younger than 65. Fifty percent of the U.S. population older than 85 has AD. Alzheimer’s is the most common form of dementia and, with diabetes, it is the 7^th^ leading cause of death in the U.S. Between 2000 and 2006, deaths because of AD increased by over 46%. It is projected that by midcentury, the number of AD patients will rise to 11-16 million [11]. 

Pathologically, AD hallmarks consist of aggregates of two abnormally folded proteins [12, 13]: beta-amyloid (Aβ) [14] and tau [15-17]. Recent data have suggested that large aggregates of these two proteins do not induce neurotoxicity, but rather small protofibrils of each may be responsible for disease pathogenesis [18-20]. In the case of amyloid, (Aβ, mostly its 42-amino acid version) accumulation occurs in the extracellular space into senile plaques. It is thought that internalization of Aβ oligomers by the neuron may be responsible for the cascade of detrimental events to the cell including tau tangle formation [21, 22]. 

## CHAPERONE-BASED THERAPIES FOR ALZHEIMER’S DISEASE AND OTHER TAUOPATHIES

Chaperones are important in tau processing and likely participate in abnormal tau accumulation [23-31]. A major component of the chaperone network is the Hsp70 family of proteins (Hsp70) that folds and degrades nascent and misfolded polypeptides [32, 33]. These can operate alone or in concert with another major chaperone protein, Hsp90. Hsp70 functions as a monomer with a single ATP-binding pocket and interacts with DnaJ proteins (Hsp40s). DnaJ proteins alter the rate of ATP consumption and play an important role in client delivery to Hsp70. Conversely, Hsp90 functions as a homo-dimer with four ATP-binding pockets and does not interact with DnaJ proteins. The higher consumption of ATP by Hsp90 has enabled more robust screening efforts that have identified inhibitors such as geldanamycin. Inhibitors of Hsp90 have been implemented in the clinic and are the first-in-class for chaperone-based therapeutics [34]. Modulators of Hsp70 have been difficult to identify because of its low intrinsic consumption of ATP. Recent work has consistently demonstrated that genetic manipulation of Hsp70, rather than Hsp90, has a considerably greater impact on the stability of proteins associated with neurodegenerative disease, including tau [26, 28, 35-38]. With this in mind, we recently worked to identify the impact of chemical modulators of Hsp70 ATPase function on tau stability [39]. 

## THE PARADOX OF CHAPERONE-BASED THERAPIES

We recently identified several compounds that either stimulate or inhibit the ATPase function of Hsp70 without inducing its expression [39-42]. These compounds generically bind to all Hsp70 family members and allosterically affect their rate of ATP consumption, which is tied to opening and closing of the protein lid over the substrate-binding domain [40]. Normally, chaperones primarily assist in protein folding and stabilization [43, 44]. But in neurodegenerative disease, where abnormal proteins accumulate, the role of chaperones may be much more diverse. Chaperones may preserve abnormal proteins rather than get rid of them, in an attempt to restore their function. Chaperones may also facilitate the degradation of misfolded disease-associated proteins they cannot repair. More recently it has been suggested that chaperones actually facilitate aggregation of disease-related proteins to prevent toxic aggregates from forming [2, 10, 45-47]. Based on these findings, much of the effort in chaperone research has focused on altering the expression levels of these heat shock proteins by activating the heat shock transcription factor (HSF1), and lead compounds have been identified that can cause this global response [48, 49]. Increasing heat shock protein levels might be expected to allow more abnormal protein clients to be bounded by the heat shock network and triaged for degradation; however, by overexpressing Hsps in several mammalian cell models of tauopathy, we have found that such increases may not be sufficient to facilitate client degradation [2]. In fact, increasing the levels of some heat shock proteins may actually preserve clients. This phenomenon suggests that there is a degree of antagonism that is built into the chaperone system [50]. Instead, inhibiting the ATPase activity of Hsp70 and Hsp90 proteins may be a much more effective strategy for dictating client fate [2, 39]. Perhaps the most effective strategy to reduce target clients would be to first increase levels of heat shock proteins, which would form more Hsp/client complexes, and then inhibit the ATPase activity of the chaperones to force client degradation, subverting any attempts at client repair.

## HSP27

Another subset of chaperones that has received limited attention from a drug discovery perspective is the small heat shock protein (sHsp) family, which consists of Hsps of molecular weight less than 30 kDa. These sHsps are conserved throughout all phyla [1], and despite structural differences, their primary role is to bind unfolded proteins and prevent them from aggregating, which creates a reservoir of intermediates for reactivation [51]. It is thought that these proteins can prevent pathological aggregation of tau, and other amyloidogenic clients [23, 52, 53]. They are unique molecular chaperones in that they function independently of ATPase activity [54]; instead, sHsps perform chaperone functions by cycling between phosphorylation-dependent oligomers and smaller-order states [55]. Among some of their functions, sHsps participate in cell survival, cytoskeletal motility, and disruption of protein aggregation [56-59]. It is the latter function that makes sHsps of great interest as a therapeutic intervention for diseases of intracellular protein aggregation like tauopathies and other neurodegenerative disorders.

Due to its particular function as a disrupter of protein aggregation [60], the Hsp of 27 kDa, Hsp27, is a recent target of interest to the field of tauopathy research. However, unlike more classical chaperones like Hsp70 and Hsp90, the current knowledge of Hsp27 function is scarce. This may be due in part to the unique nature of Hsp27 as an ATPase-independent chaperone [54], which inherently makes it difficult to establish functional assays. Furthermore, Hsp27’s function is determined by a dramatic, phosphorylation-dependent change in quaternary structure; this dynamism makes the elucidation of structure-function relationships very challenging. During quiescent conditions, Hsp27 mostly exists as a large oligomeric conformer of 200kDa-800kDa [61, 62]. Upon a stress response, it becomes phosphorylated at three serine sites (S15, S78, and S82). As a result, phosphorylated Hsp27 breaks apart into a smaller conformation of monomers, dimers, and tetramers that allow scavenging of misfolded polypeptides favorable. It is presumed that clients interact with the smaller assemblies of Hsp27 [51, 60, 63, 64]. Next, the client-bound Hsp27 resurges into a large oligomeric complex while still bound to client, as suggested by studies in other sHsps [60, 65, 66]. Experimental evidence using Hsp27 mutants retained in either pseudo-phosphory-lated or perpetually dephosphorylated conformations suggests that it is the latter assembly that results in chaperone function. Albeit these results, recent evidence from our laboratory suggest that the processes are more complex in that the dynamic cycling from small to large structures is necessary for proper Hsp27 chaperone function in the dissolution of toxic protein aggregates [10].

Hsp27 is the human heat shock protein of 27 kilodaltons, which is encoded by a single intronless gene termed *HSPB1*. The mouse homolog is Hsp25. The gene resides on chromosome 7q11.23, and mutations in it have been associated with Charcot-Marie Tooth syndrome, a distal motor and sensory neuropathy that is caused by mis-aggregation of the Hsp27 protein itself [67]. Both the amino- and carboxy-termini of Hsp27 allow it to interact with other proteins [62]. The carboxy-terminus of all sHsps, including Hsp27, is characterized by having a conserved region that is related to the vertebrate lens protein α-crystallin [68, 69]. Hsp27’s α-crystallin domain is located between residues 87 and 167 [68]. In 1982, Ingolia and Craig hypothesized that since α-crystallins in the lens form soluble multimers then this shared domain would also facilitate Hsp27 oligomerization Ingolia and Craig 1982 PNAS). Indeed, experimental evidence using site-directed spin labeling indicated that the α-crystallin domain of Hsp27 is critical for the formation of discreet oligomeric structures with symmetrical orientation [70]. A second, less conserved region of all sHsps is the WDPF domain. It is characterized by a conserved tryptophan-aspartate-proline-phenylalanine sequence, and it lies at the N-terminus. Unlike the α-crystallin domain, the WDPF domain is sensitive to phosphorylation, and this process is critically linked to the multimerization of Hsp27 [62]. 

Functional modulation of human Hsp27 is triggered by phosphorylation at three key sites (S15, S78, and S82) by the p38 mitogen-activated protein kinase mitogen-activated protein kinase (MAPK)-activated protein kinase 2 and Akt pathway (for a thorough review see [71]). Phosphorylation of Hsp27 is reversible and dephosphorylation is mainly attributed to protein phosphatase 2A [72]. The result of cycling between phosphorylated and dephosphorylated states causes Hsp27 to rearrange from smaller assemblies to large self-aggregated rearrangements. This dynamic process results in a dual and independent function of Hsp27, both as a regulator of actin microfilament dynamics and as a molecular chaperone that prevents unfolded protein aggregation [62] Fig. (**1**). 

Before its identification, Hsp27 had been functionally defined as an inhibitor of actin polymerization in smooth muscle of turkey gizzards [73, 74]. It was later found that stress-induced actin depolymerization was partially blocked in CHO cells over-expressing Hsp27 [75]. This relationship was further evaluated using CHO cells over-expressing either wild-type Hsp27 or an Hsp27 mutant that is phosphorylation incompetent: only wild-type Hsp27 could stabilize actin filaments under stress [76], Thus it can be suggested that Hsp27 phosphorylation is a critical process for Hsp27 function. This finding was also corroborated by a time-course study in thrombin-activated platelets showing that unbound, cytoplasmic Hsp27 must be phosphorylated before it can attach to actin [77]. Phosphorylation kinetics are not responsible for Hsp27 cellular localization, but they are required for regulating microfilament formation [78, 79]. It was determined that only unphosphorylated, monomeric Hsp27 is able to block actin polymerization [80], and therefore manipulation of Hsp27 phosphorylation state enables genetic regulation of pinocytosis [76], cell migration [81-83], and muscle contraction [73, 74], among many others. 

The other major role of Hsp27 is that of a molecular chaperone; however, the mechanism contributing to this activity is less clear. To date, it is thought that only large multimeric Hsp27 complexes are capable of chaperone activity [55]. This is largely based on evidence that substrates/clients have previously been detected attached to Hsp27 oligomers [51, 60, 63, 64]. The ultimate fate of the bound client is likely driven by interactions with specific co-chaperones and the extent of damage to the misfolded client. Data from *in vivo* and *in vitro* experiments show that Hsp27 can prevent self-association of aggregation prone polypeptides like the insulin B chain upon disulphide reduction, malate dehydrogenase, and citrate synthase, among others [51, 63, 84]. In addition, Hsp27 can also hold misfolded or intrinsically disordered clients such as tau in a conformation that is stable for subsequent transfer to Hsp70, culminating in client refolding at the expense of ATP [51]. Hsp27 can also deliver clients for ubiquitin-independent clearance via the proteasome in a mechanism that is not well characterized [23], but it likely involves other chaperones such as Hsp90 and the ubiquitin ligase CHIP [24] Fig. (**2**). It is this capacity of Hsp27 to hold and prevent aggregation of unfolded clients that is interesting for neurodegenerative disorders like tauopathies, synucleinopathies, and poly-glutamine diseases, where aggregation of misfolded proteins is the confounding pathogenic hallmark. Thus, enhancing Hsp27 levels or function to prevent aggregation of these toxic elements, may serve as a potential therapeutic strategy in neurodegenerative diseases.

## HSP27 EXPRESSION AND LOCALIZATION IN THE CNS IS SITE- AND CELL-SPECIFIC

Hsp27 is detectable under basal conditions in both a site and cell-specific manner in the central nervous system (CNS). The patterns of Hsp27 expression following stress and during development also adhere to specific anatomical patterns. Moreover, differentiating cells are Hsp27 immuno-reactive, while apoptotic cells lack Hsp27 expression [85]. This suggests that Hsp27 participates in cell survival during development and into adulthood [4, 6, 86, 87]. 

Under basal conditions, Hsp27 immuno-reactivity is scarce but detectable in motor and sensory neurons of the spinal cord and brain stem [88], most ocular neural structures [89], and a small set of Purkinje cells in mouse cerebellum [90-92]. Levels of constitutive Hsp27 are highest in astrocytes surrounding leptomeningeal and parenchymal blood vessels [93]. Meanwhile, other brain regions are virtually devoid of constitutive Hsp27 immuno-reactivity, and if found, it’s mostly in glial populations [93-99]. 

Site-specific up-regulation of Hsp27 is detected upon stimulation by various stress triggers. For instance, while Hsp27 is barely detectable in the cortex and hippocampus of un-injured rats [88, 98], it is induced in these regions upon stressors like hyperthermia and ischemia [96-98]. Hyperthermia also stimulates Hsp27 expression in large glial populations and some neurons of forebrain, brain stem, hypothalamus, and cerebellum [100-102]. Other examples of stress-related Hsp27 inducers include; middle cerebral artery occlusion [97, 100, 101], traumatic nerve injury [103-106], and kainic acid-induced epilepsy [95, 99, 100, 102]. 

## HSP27 AND AD

In addition to the stressors described above, Hsp27 is also increased in the brains of AD patients [107-109]. This phenomenon could be explained by the convergence of a variety of stress insults that occur in the AD brain, including oxidation, inflammation, or aberrant calcium influx [110, 111]. This up-regulation of Hsp27 appears to be site and cell-specific. For instance, using immuno-histochemical, sandwich ELISA, and dot blot analyses, several groups determined that AD brains express significantly increased levels of Hsp27 in the frontal, parietal, and temporal cortices, but not in occipital cortex when compared to age-matched non-demented controls; specifically, Hsp27 co-localized with areas affected by senile plaques and cerebral amyloid angiopathy [53, 93, 107, 109]. Constitutive Hsp27 expression in control brains was mostly found in glia surrounding leptomeningeal and parenchymal blood vessels and occasionally in a scarce number of astrocytes throughout the cortex of control brains, as described above [107]. 

This distribution suggests that efforts to increase the levels of Hsp27 in neurons may be a neuro-protective strategy in AD [95-99]. For example, one family of compounds that specifically upregulates Hsp27 expression without affecting the expression of other Hsps is the statins [112-118]. Indeed statins are proposed to have therapeutic efficacy for AD [119-123] and increasing Hsp27 levels may be one of several mechanisms that are beneficial for tau-bearing neurons. Thus identifying therapeutics that specifically upregulate Hsp27 in neurons may reduce the extent of neuronal damage, prevent neuronal death, and increase neuronal function despite neurotoxic stressors. As a counterpoint to this approach, it is important to consider the possible negative consequences to the neuron that result from sustained up-regulation of Hsp27 protein levels. Perpetual induction of Hsp27 may ultimately be detrimental over time. Perhaps intermittent delivery strategies that temporally upregulate Hsps like Hsp27 may be the most sustainable and effective way to target these proteins therapeutically. 

## HSP27 AND Aβ

The capacity of Hsp27 to ameliorate amyloid β (Aβ)-induced toxicity has been elegantly evaluated in a series of *in vitro* experiments [94]. Primary cortical neurons of P1 rats over-expressing Hsp27 showed reduction in Aß-mediated cell death, increased neurite outgrowth, and mitochondrial protection from Aß-induced damage [94]. Importantly, only neuronal Hsp27 conferred survival; changes to glial or endothelial Hsp27 levels were not protective. These results corroborate previous work showing that Hsp27 could abrogate Aβ aggregation and toxicity [124-126]. Three independent studies show that Hsp27 binds Aβ, whether it be in the form of Aβ_40_ [126], Aβ_42_ [94], or a particular variant of Aß_40_ that carries the Dutch mutation (D-Aβ_40_) [124]. Aggregation of the different Aß subtypes was also decreased upon incubation with Hsp27, except when Aß_42_ fibrils formed [125]. Together, these results suggest that Hsp27 binds aggregation-prone Aβ, reduces its ability to assemble into fibrils, and consequently confers protection from cell death.

## HSP27 AND TAU

In addition to Aß, Hsp27 also associates with tau tangles in AD, as well as other tauopathies including progressive supranuclear palsy, corticobasal degeneration and fronto-temporal dementia [53, 127, 128]. Interestingly, while biochemical assays show increased total levels of Hsp27 in AD, immuno-staining assays reveal that the bulk of Hsp27 (apart from that found in tangles) is found in glia, particularly astrocytes. 

In 2004, Shimura *et al*. more thoroughly evaluated the interaction of Hsp27 with tau [23]. Using human brain lysates from both AD and age-matched control individuals, they identified Hsp27 as a direct binding partner of phosphorylated tau. These results were later corroborated in larger studies of AD brains, where Hsp27 levels were shown to correlate with neurofibrillary tangle pathology progression [53, 127, 128]. The interaction between Hsp27 and tau was enhanced by phosphorylation of either protein [23]. While these findings suggest that Hsp27 preferentially interacts with aberrant tau, the persistence of tangles in the brain of AD patients despite this interaction suggests that Hsp27 alone is not sufficient to prevent tau pathogenesis.

Subsequent experiments in human neuronal-like cells revealed that both Hsp27 and its phosphorylated form, pHsp27, participated in the dephosphorylation of tau and perhaps facilitated tau degradation [23]. Cell culture studies showed that both wildtype and phosphorylated Hsp27 were able to prevent tau aggregation and prevent apoptosis [23]. These results further suggest that Hsp27 is capable of successfully preventing pathogenic protein aggregation and attenuating toxicity. But this protection is only imparted during stress. Therefore, chemical or genetic induction of neuronal Hsp27 could be exploited to protect neurons from the adverse consequences of intra-neuronal tangle accumulation. 

## CONCLUDING REMARKS

Although *in vitro* and *in vivo* experimental efforts have yielded successful abrogation of AD pathology by inhibiting Hsp70 and Hsp90, the translation of these approaches to human therapeutics may lead to complications in many other biological processes linked to the normal functioning of these proteins, and therefore lead to undesirable outcomes. As such, Hsp70 and Hsp90 inhibition become unattractive therapeutic targets. However, such experimental success paves the road to focus on Hsps as powerful tools to combat diseases rooted in the abnormal aggregation of proteins in cells. A shift of attention to explore the family of sHsps has procured data highlighting the impact of Hsp27 on preventing disease progression [10]. 

Any therapeutic strategy aimed at up-regulating Hsp27 levels must override the cell’s natural tendency to express low levels of endogenous Hsp27. For instance, the murine leukemic cell line P388 is incapable of expressing endogenous Hsp25 despite heat shock stimulation [129]. It is possible that P388 cells have an Hsp25 gene silencing mechanism since HeLa cells transfected with the P388 Hsp25 promoter were capable of inducing expression. Whether neurons are under similar regulatory constraints that subdue Hsp27 expression remains to be determined. However, this regulation of Hsp27 is not confined to all neurons. In fact, Hsp27 induction is observed after ischemic stress in rat neurons of the fourth and fifth cortical layers and pyramidal neurons in the hippocampus [97]. Besides endogenous production as a primary means for Hsp27 upregulation, it would be interesting to determine if these neurons are capable of internalizing glial-Hsp27 therefore exploring a secondary mechanism for increased neuronal Hsp27. Other possible means to enhance Hsp27 levels could include slowing neuronal Hsp27 turnover or even extrinsically delivering recombinant sHsps derived from other organisms. It is possible that these homologous versions of Hsp27 may have even more potent activity for reducing aggregation than human Hsp27 itself Fig. (**3**). 

Further understanding of the mechanisms regulating neuronal Hsp27 expression will provide insight for the development of new therapies. A neuron-specific strategy may be the most effective for treating neurodegenerative tauopathies, the most common of which is AD. Furthermore, this review has focused on only one of the many members of the large and conserved family of sHsps. The complex regulation of this phylogenetically conserved group of proteins may coordinate their activities to clear the pathological inclusions associated with AD. Defining these mechanisms will likely lead to improved rational drug design for targeting proteotoxic diseases.

## Figures and Tables

**Fig. (1):**
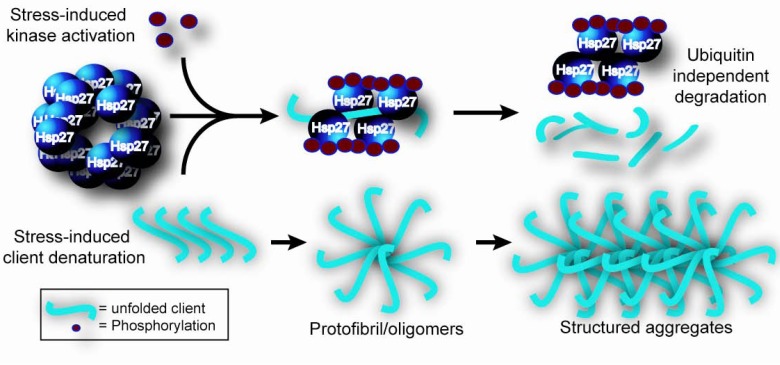
The necessity of phosphorylation-dependent Hsp27 function for preventing protein aggregation of unfolded intermediates. Large molecular weight Hsp27 multimers are constitutively present in the cell under basal conditions. Upon extracellular stress, the p38 MAP kinase pathway is activated, phosphorylating Hsp27 (red circles). Consequently, the Hsp27 multimeric complex disassembles into smaller complexes that can bind the denatured client and prevent its subsequent aggregation. The client can then be targeted for degradation in a mechanism that is independent of ubiquitin. Without Hsp27, folding intermediates of the client would be prone to aggregate.

**Fig. (2):**
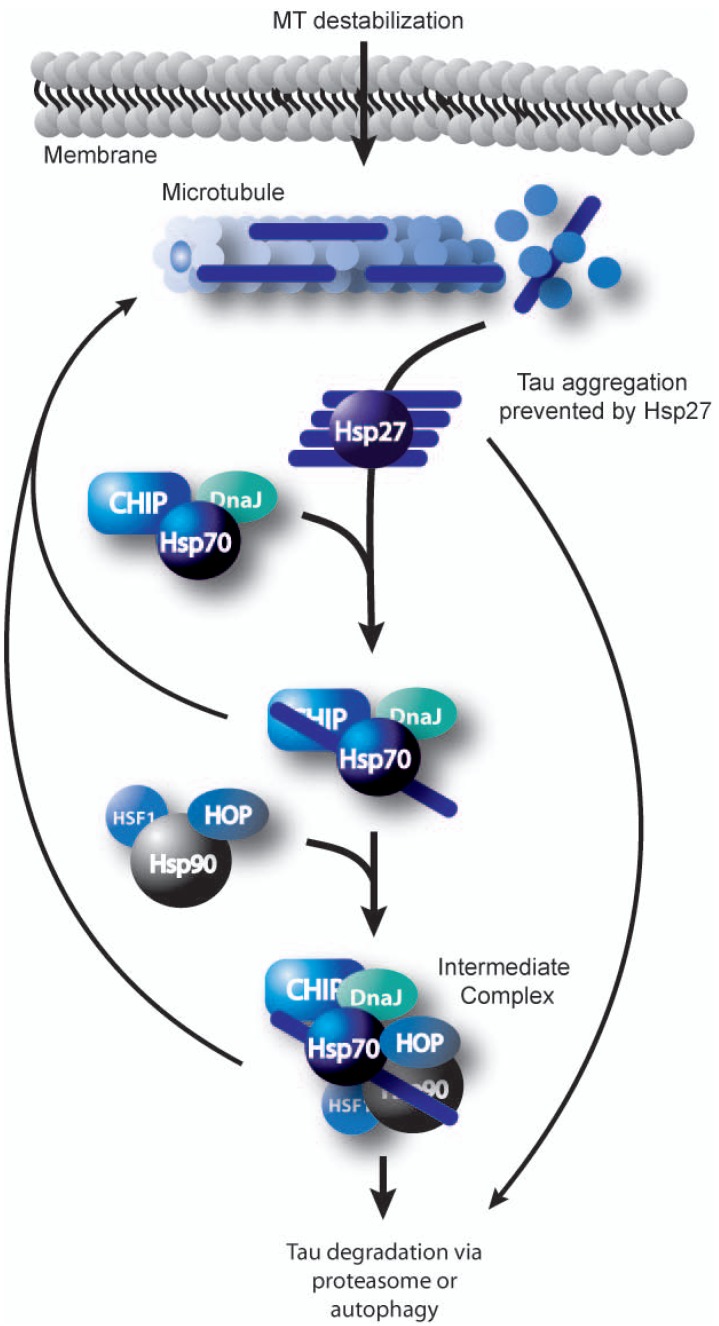
Coordination of substrate transport through the chaperone network. Upon stress induced microtubule disassembly, Hsp27 and perhaps other small Hsps, bind tau to prevent its toxic aggregation. The Hsp27/tau complex is stable, and tau can then be transferred to the Hsp70/DnaJ chaperone complex. If refolding is not possible, the tau-bound Hsp70 complex will associate with the Hsp90 complex for further attempts at refolding. Alternatively, the client is processed for degradation. The decisions are made based on coordinate regulation by the chaperones and cochaperones associating with the complex, such as Hsp90, CHIP and Hop.

**Fig. (3):**
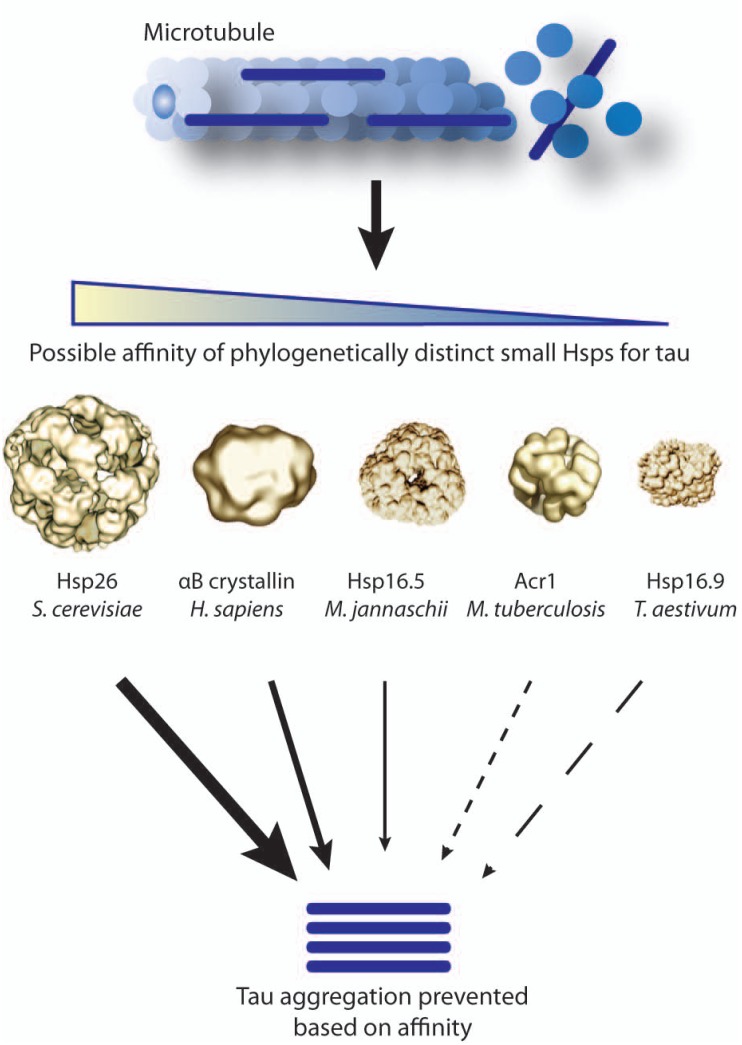
The variable affinity of phylogenetically diverse sHsps for tau may be therapeutically relevant. Client specificity of sHsps is not well characterized. Small Hsp-mediated prevention of toxic tangle formation can be further enhanced by selecting sHsps, perhaps even from other species, with higher affinity for tau and driving their expression. Therefore certain sHsps will likely bind tau quicker and more stably, intervening before tau can begin to aggregate.

## ABBREVIATIONS

Hsp: = Heat shock proteins
sHsp: = Small Hsp
AD: = Alzheimer’s disease
